# Role of Adipokines in Atherosclerosis: Interferences with Cardiovascular Complications in Rheumatic Diseases

**DOI:** 10.1155/2012/125458

**Published:** 2012-07-15

**Authors:** Morena Scotece, Javier Conde, Rodolfo Gómez, Verónica López, Jesús Pino, Antonio González, Francisca Lago, Juan J. Gómez-Reino, Oreste Gualillo

**Affiliations:** ^1^SERGAS, Santiago University Clinical Hospital and NEIRID Lab (NeuroEndocrine Interaction in Rheumatology and Inflammatory Diseases), Institute of Medical Research (IDIS), Building C, Level-2, 15706 Santiago de Compostela, Spain; ^2^Division of Rheumatology, Fundación Jiménez Diaz, 2-28040 Madrid, Spain; ^3^SERGAS, Santiago University Clinical Hospital and Division of Orthopaedics Surgery and Traumatology, 15706 Santiago de Compostela, Spain; ^4^SERGAS, Santiago University Clinical Hospital and Laboratory of Genetics in Rheumatic Diseases, Institute of Medical Research (IDIS), Building C, Level-2, 15706 Santiago de Compostela, Spain; ^5^SERGAS, Santiago University Clinical Hospital and Research Laboratory 7 (Molecular and Cellular Cardiology), Institute of Medical Research (IDIS), 15706 Santiago de Compostela, Spain

## Abstract

Patients with rheumatic diseases have an increased risk of mortality by cardiovascular events. In fact, several rheumatic diseases such as rheumatoid arthritis, osteoarthritis, systemic lupus erythematosus, and ankylosing spondylitis are associated with a higher prevalence of cardiovascular diseases (CVDs). Although traditional cardiovascular risk factors have been involved in the pathogenesis of cardiovascular diseases in rheumatic patients, these alterations do not completely explain the enhanced cardiovascular risk in this population. Obesity and its pathologic alteration of fat mass and dysfunction, due to an altered pattern of secretion of proinflammatory adipokines, could be one of the links between cardiovascular and rheumatic diseases. Indeed, the incidence of CVDs is augmented in obese individuals with rheumatic disorders. Thus, in this paper we explore in detail the relationships among adipokines, rheumatic diseases, and cardiovascular complications by giving to the reader a holistic vision and several suggestions for future perspectives and potential clinical implications.

## 1. Introduction

Patients with rheumatic diseases have an increased risk of mortality and fatal cardiovascular events. Several rheumatic diseases including rheumatoid arthritis (RA), osteoarthritis (OA), systemic lupus erythematosus (SLE), and ankylosing spondylitis (AS) have been associated with higher prevalence of cardiovascular diseases (CVDs) [1]. For instance, CVDs are responsible for almost 50% excess of mortality in patients with RA [2, 3].

Classic risk factors such as obesity and the related metabolic syndrome, presented in patients with rheumatic diseases, might explain the increased risk of CVDs occurred in rheumatic disorders [4]. In fact, there are reports showing a major prevalence of metabolic syndrome in lupus patients compared to healthy controls, and a higher risk of CVDs in these patients was also reported [5, 6]. Moreover, it has been reported that there is a considerably higher prevalence to develop metabolic syndrome and CVDs in AS patients [7].

White adipose tissue is described as an endocrine organ, which secrete a wide variety of factors called adipokines, which have multiple functions. At present, it is well known that adipokines play relevant roles in the pathophysiology of rheumatic diseases and CVDs [8, 9]. To note, visceral fat accumulation associated with adipokine dysregulation affects both atherosclerotic plaque development and plaque disruption [10, 11]. Clearly, when the advanced plaque becomes unstable, ruptures can occur, establishing an acute coronary syndrome that is aggravated by the adipokine-induced prothrombotic and inflammatory state, which can further worsen syndromes.

Here, we present an updated review based on the function played by four adipose tissue-derived factors (leptin, adiponectin, visfatin, and resistin) in atherosclerosis and different rheumatic diseases.

## 2. Leptin

Leptin is a 16 kDa nonglycosylated hormone encoded by the gene *ob*, the murine homologue of the human gen *LEP* [12]. It belongs to class I cytokine superfamily, consisting of a bundle of four *α*-helices. Leptin is mainly produced by adipocytes and circulating levels are correlated with WAT (white adipose tissue) mass. It decreases food intake and increases energy consumption by acting on specific hypothalamic nuclei, where leptin induces anorexigenic factors as cocaine amphetamine-related transcript (CART) and suppressing orexigenic neuropeptides such as neuropeptide Y (NPY) [13]. Leptin levels are mostly dependent on the amount of body fat, but its synthesis is also regulated by inflammatory mediators [14]. Leptin exerts its biological actions through the activation of its cognate receptors, which are encoded by the diabetes gene (*db*) and belong to the class I cytokine receptor superfamily. There are six different isoforms of leptin receptors, but only the long isoform is functional (Ob-Rb). Several tissues produce leptin and express its receptors, including those of the cardiovascular system such as blood vessels and cardiomyocytes [15]. Leptin gene expression is mainly regulated by food intake, energy status, hormones, and also by inflammatory mediators [8, 16]. Genetic deficiency in the gene encoding for leptin or its receptors provokes severe obesity and diabetes mellitus.

## 3. Leptin and Atherosclerosis

**Figure 1:**
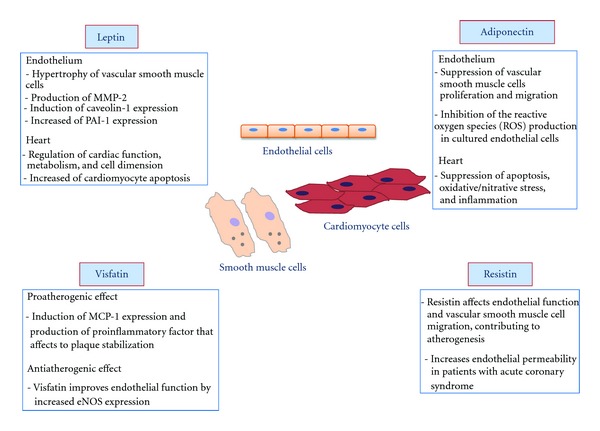
Schematic representation of the involvement of leptin, adiponectin, resistin, and visfatin in atherosclerosis.

Leptin is associated with cardiovascular diseases (CVD) (Figure 1), In fact, elevated serum concentrations of this adipokine are related with myocardial infarction and stroke independently of traditional cardiovascular risk factors [17]. Moreover, it has been proposed that leptin plays a pathogenic role in atheromatous plaques, due to its positive association with C-reactive protein (CRP) and soluble IL-6 receptor (sIL-6R) [18], two inflammatory mediators involved in the pathogenesis of atherosclerosis [19, 20]. The proatherogenic actions of leptin are supported by several experimental observations demonstrating that this adipokine induces hypertrophy of vascular smooth muscle cells [21] and the production of matrix metalloproteinase 2 (MMP-2) [22]. The latter develops main actions in plaque rupture [23]. Also, leptin could stimulate vascular remodeling by promoting profibrotic cytokines production [24]. Apart from this, leptin increases the secretion of proatherogenic lipoprotein lipase by cultured human and murine macrophages [25], enhances platelet aggregation [26, 27], and induces C-reactive protein (CRP) expression in human coronary artery endothelial cells [28].

It has been described that leptin induces caveolin-1 expression in endothelial cells [29]. Caveolin-1 plays a relevant regulatory role in the development of atherosclerosis, promoting the transcytosis of LDL to the subendothelial space, and inhibiting eNOS function [30, 31]. This study represents a novel mechanism through which hyperleptinemia contributes to the development of atherosclerosis. Recently, it has been reported that leptin was able to increase plasminogen activator inhibitor-1 (PAI-1) expression in human coronary artery endothelial cells [32]. PAI-1 plays an important role in the development and progression of atherosclerosis [33, 34], with its deficiency described to protect being against atherosclerosis progression [35]. Indeed, in human atherosclerotic arteries, PAI-1 production and enhanced expression appear to be directly related with the degree of atherosclerosis [36].

## 4. Leptin and Rheumatic Diseases

In addition to its well-known actions on immune system, leptin has also been associated with rheumatic diseases due to its ability to modulate bone and cartilage metabolism [37, 38].

Leptin plays main actions in certain autoimmune diseases such as rheumatoid arthritis (RA). This idea is supported by several *in  vitro* and *in  vivo* studies. Serum leptin levels were increased in RA patients compared to healthy controls [39, 40]; however, other studies reported unchanged levels [41]. Moreover, several authors suggested that a correlation between the RA disease activity and leptin levels might exist [42–44]. To note, synovial/serum leptin ratio was correlated with disease duration and erosion parameters in RA patients [45], whereas other authors did not found any correlation between leptin levels and disease activity [46]. In patients undergoing anti-TNF-*α* therapy because of severe diseases refractory to conventional therapy, there was a positive correlation between body mass index of RA patients and serum level of leptin [46]. Interestingly, in these patients there was a correlation between leptin levels and VCAM-1 [46]. This is of potential irrelevance as biomarkers of endothelial dysfunction endothelial cell activation have been found elevated in patients with RA and anti-TNF blockade improved endothelial dysfunction [47] and also yielded a decrease of the levels of some of these endothelial cell activation biomarkers [48]. Regrettably, although different studies have confirmed the influence of gene polymorphisms, located in inside and outside the MHC region, in the increased risk of endothelial dysfunction and cardiovascular events observed in patients with RA [49–51], leptin-LEP polymorphisms do not seem to be a genetic risk factor for disease susceptibility or clinically evident cardiovascular disease and subclinical atherosclerosis in patients with RA [52].

Low leptin levels, related with food restriction, have been linked to CD4+ lymphocyte hyporeactivity and increased interleukin-4 secretion [53]. Leptin was involved in RA-induced hypoandrogenicity, due to the fact that leptin levels were negatively correlated to androstenedione [54]. Then, since leptin acts as a proinflammatory factor and androgens are commonly considered as anti-inflammatory agents, the preponderance of leptin and hypoandrogenicity may help to perpetuate chronic rheumatic diseases such as RA. In addition, TNF-*α* blockers such as infliximab or adalimumab did not modify serum leptin levels [46, 54, 55]. Several studies carried out in arthritis animal models, as well as *in  vitro* studies, support the involvement of leptin in RA [56].

Leptin stimulation increases IL-8 production in RA synovial fibroblasts via leptin receptor/JAK2/STAT3 pathway [57]. However, the effects of leptin in RA are not only related to articular tissues. Leptin also modulates the activity of multiple immune cells, including regulatory T cells, which are potent inhibitors of autoimmunity [58]. The ability of leptin to induce regulatory T cells anergy and T-cell receptor hyporesponsiveness has gained much interest since altered functioning of this cell type was described in RA [59].

Leptin has also been related with osteoarthritis (OA) and cartilage metabolism. It is known that chondrocytes from human OA cartilage produce much more leptin than chondrocytes from normal cartilage [60]. Moreover, leptin was found in synovial fluid from OA-affected joints [60, 61]. In fact, the expression pattern of leptin was related to the grade of cartilage destruction [60] and with the severity of the disease, with the highest levels of leptin in being the advanced stages of the disease [62, 63].

Recently it has been reported that extreme obesity due to the impaired leptin signalling induces alterations in subchondral bone morphology but without increasing the incidence of OA [64]. These results suggest that obesity, per se, is not sufficient to induce OA, leptin being necessary in the development and progression of OA associated with obesity. *In  vitro* experiments also pointed a role of leptin in OA. Leptin increases IL-8 production by OA synovial fibroblasts and chondrocytes [57, 65]. In human cultured chondrocytes, leptin synergizes with IL-1 and interferon-*γ* in the synthesis of nitric oxide [38, 66]. In addition, this adipokine enhances MMP-9, MMP-13, prostaglandin E_2_ and IL-6 production in human chondrocytes [63, 67]. Leptin has also been related with bone metabolism. Actually it has been suggested that abnormal leptin production by OA osteoblasts could be responsible for an altered osteoblast function in OA [68].

Regarding the role of leptin in systemic lupus erythematous (SLE), some contradictory data exists. Nowadays, most of the studies suggest a role for leptin in this disease. Several authors found higher leptin levels in SLE patients compared with healthy controls, even after BMI correction [69–73]. Interestingly, in some of these studies, the hyperleptinemia was associated with cardiovascular diseases and with several features of the metabolic syndrome [72, 73]. Indeed, using a lupus animal model, it was determined that leptin enhanced the proinflammatory high-density lipoproteins scores and atherosclerosis induced by a high-fat diet [74], indicating that factors related with metabolic syndrome can accelerate the disease and its cardiovascular complications. On the other hand, other groups have described lower or unchanged circulating leptin levels in SLE patients compared to healthy control [75, 76].

**Figure 2:**
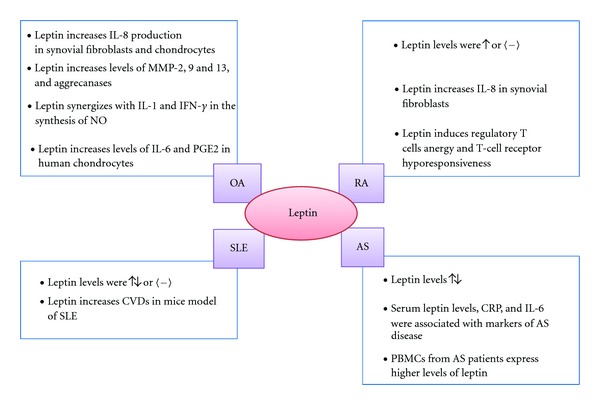
Schematic representation of leptin implication in several rheumatic diseases.

The role of leptin in ankylosing spondylitis (AS) is still unclear and the data available are almost limited. For instance, certain studies have not found any correlation between serum leptin concentrations and markers of disease activity [77, 78]. However, other authors determined an association among serum leptin levels, CRP, IL-6, and markers of disease activity [79, 80]. In addition, it has been also reported that peripheral blood mononuclear cells (PBMCs) from AS patients express higher amounts of leptin compared with PBMCs from controls [81], and exogenous administration of leptin exacerbates the production of proinflammatory cytokines in PBMCs from AS patients compared with those from controls [81] (Figure 2).

## 5. Adiponectin

Adiponectin, also known as GBP28, apM1, Acrp30, or AdipoQ, is a 244-residue protein with structural homology to types VIII and X collagen and complement factor C1q that is prevalently synthesized by adipose tissue. Adiponectin circulates in the blood in large amounts and constitutes approximately 0.01% of the total plasma proteins and it is secreted from adipocytes as different molecular forms (trimers, hexamers, and also 12–18-monomer forms) [82, 83]. It increases fatty acid oxidation and reduces the synthesis of glucose in the liver and other tissues [82]. Ablation of the adiponectin gene has no dramatic effect on knockout mice on a normal diet, but when placed on a high-fat/sucrose diet, animals develop severe insulin resistance and exhibit lipid accumulation in muscles [84]. Circulating adiponectin levels tend to be low in morbidly obese patients and increase with weight loss and with the use of thiazolidinediones (insulin-sensitizing drugs) which enhance sensitivity to insulin [82, 85].

Adiponectin acts via two receptors, one (AdipoR1) found predominantly in skeletal muscle and the other (AdipoR2) in liver. Transduction of the adiponectin signal by AdipoR1 and AdipoR2 involves the activation of AMPK, PPAR-*α*, PPAR-*γ*, and other signalling molecules [82]. To note, targeted disruption of AdipoR1 and AdipoR2 causes abrogation of adiponectin binding and all its metabolic actions [86]. The gene that codes for human adiponectin is located on chromosome 3q27, a locus linked with susceptibility to diabetes and cardiovascular diseases [87].

## 6. Adiponectin and Atherosclerosis

Unlike most other adipokines, plasma levels of adiponectin are decreased in obesity and related pathologies, including type 2 diabetes and cardiovascular diseases [88]. Adiponectin possesses multiple healthy effects on obesity-related metabolic complications, dyslipidaemia, nonalcoholic fatty liver disease, and several types of cancers [89]. It has been suggested that hypoadiponectinemia is an independent risk factor for hypertension [90] and has a detrimental effect on aortic stiffness [91]. Furthermore, subjects carrying the genetic variants that are related to lower plasma levels of adiponectin have a higher risk of hypertension [92, 93]. Several studies have shown that dyslipidemia is also associated with low circulating levels of adiponectin, even in the absence of other metabolic syndrome risk factors [94]. Hypoadiponectinemia has been linked to inflammatory atherosclerosis, suggesting that normal adiponectin levels are required to maintain a noninflammatory phenotype on the vascular wall [8].

Adiponectin might regulate many steps in the atherogenic process, such as antiapoptotic actions on endothelial cells and angiogenic effects on the vasculature [95]. Antiatherosclerotic effects of adiponectin were exerted through multiple actions on almost each vascular cell type, such as cardiomyocyte endothelial cell and endothelial progenitor cell. Particularly, adiponectin inhibits neointimal formation by suppressing proliferation and migration of vascular smooth muscle cells [96–98], blocks inflammation and foam cell formation from macrophages [99, 100] and stimulates the production of the anti-inflammatory cytokine IL-10 and of interleukin 1 receptor antagonist (IL1Ra) by macrophages [101]. Adiponectin also was able to inhibit the production of reactive oxygen species (ROS) in cultured endothelial cells [102–104]. In addition to its effects on the vasculature, several studies *in  vitro* and *in  vivo* demonstrated that adiponectin acts directly on cardiomyocytes to protect the heart from ischaemic injury, hypertrophy, cardiomyopathy and systolic dysfunction [105]. In particular, the cardioprotective effects of adiponectin are attributed to its ability in suppressing apoptosis, oxidative/nitrative stress, and inflammation in cardiomyocytes [106]. Also, high plasma adiponectin levels are associated with a lower risk of myocardial infarction in men [107], a reduced coronary heart disease risk in patients with diabetes mellitus [108], and a lower risk of acute coronary syndrome [109] (Figure 1).

## 7. Adiponectin and Rheumatic Diseases

In contrast to its previously described protective role in cardiovascular diseases and obesity, there are multiple evidence that adiponectin acts as a proinflammatory factor in joints and it could be involved in matrix degradation. Adiponectin levels have been found to be higher in RA patients than in healthy controls [39, 110–114]. Recently, it has been reported that adiponectin and adiponectin receptor-1 expression are higher in synovial fluids and the synovial tissues of RA patients compared with controls, confirming the correlation of circulating adiponectin levels with the severity of RA [115]. In RA patients undergoing anti-TNF infliximab therapy because of severe disease, high-grade inflammation was independently and negatively correlated with circulating adiponectin concentrations, whereas low adiponectin levels clustered with metabolic syndrome features such as dyslipidemia and high plasma glucose levels that have been reported to contribute to atherogenesis in RA [116]. However, the interaction of high-grade inflammation with low circulating adiponectin concentrations does not likely to be TNF-*α* mediated in RA [116]. Also, no association between adiponectin and carotid intima-media wall thickness, a surrogate marker of cardiovascular events in RA [117], was observed in patients with RA [118]. In keeping with these negative results, no associations between functional adiponectin-ADIPOQ rs266729 and ADIPOQ rs1501299 polymorphisms and cardiovascular disease were found in patients with RA [119].

Several studies supported the catabolic role for adiponectin. It has been reported that adiponectin is able to stimulate the production of PGE2, IL-6, IL-8, vascular endothelial growth factor, and MMP-1 and MMP-13 in RA synovial fibroblasts [62, 120–122]. In addition, in cultured human chondrocytes and synovial fibroblasts, adiponectin also induces the production of NO, IL-6, MMP-3, MMP-9, monocyte chemotactic protein 1 (MCP-1), and IL-8 [65, 123–125]. Adiponectin has a similar behaviour in other cell types also involved in the RA, such as lymphocytes and human macrovascular endothelial cells. This adipokine promotes inflammation through increased TNF-*α*, IL-6, IL-8, and RANTES secretion by human primary lymphocytes. Moreover, it induces IL-6, IL-8, MCP-1, and RANTES secretion by human macrovascular endothelial cells [126, 127].

Concerning the role of adiponectin in SLE, several studies have showed elevated levels of this adipokine in SLE patients [70, 73, 75]. Nevertheless, other authors did not find any difference in adiponectin levels between SLE patients and controls [72, 128]. However, the same authors find a positive correlation of leptin with vascular stiffness parameters whereas adiponectin inversely correlates [129].

In the study by Rovin et al. [130], the authors showed that serum adiponectin levels are higher in patients with renal SLE than in healthy controls and in patients with nonrenal SLE. In addition, lower levels of adiponectin were presented in SLE patients with insulin resistance (IR) compared to SLE subjects without IR [70]. It also has reported that mice with experimental lupus, that lack adiponectin, develop more severe disease than wild-type mice, suggesting the involvement of adiponectin in regulating disease activity [131].

In addition, very recently, McMahon and colleagues have demonstrated that leptin levels confer increased risk of atherosclerosis in women with systemic lupus erythematosus and that there is no significant association between adiponectin and atherosclerotic plaques in SLE [132].

**Figure 3:**
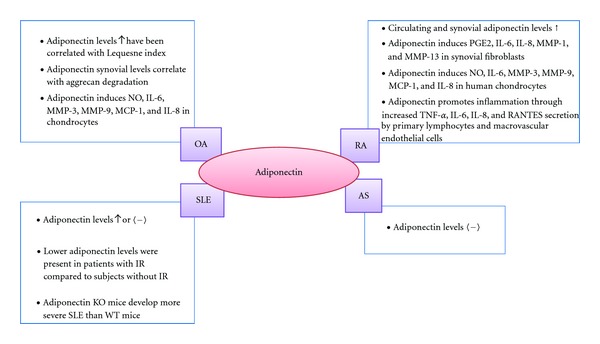
Schematic representation of adiponectin implication in several rheumatic diseases.

Little is known about the role of adiponectin in other rheumatic diseases, such as AS and Sjögren's syndrome. However, it has reported that serum adiponectin levels are not different between AS patients and healthy controls [78]. Regarding the Sjögren's syndrome, it has been described that adiponectin is expressed in salivary gland epithelial cells, and this expression is higher in patients with Sjögren's syndrome [133]. Moreover, the same group was demonstrated that adiponectin is able to protect salivary gland epithelial cells from spontaneous and INF-*γ*-induced apoptosis [134] (Figure 3).

## 8. Visfatin

Visfatin, also called PBEF (pre-B-cell colony-enhancing factor), and Nampt (nicotinamide phosphoribosyltransferase), is a protein of approximately 471 amino acids and 52 kDa [135]. It is a hormone that originally was discovered in liver, bone marrow, and muscle, but it is also secreted by visceral fat [135, 136].

It has been reported that visfatin is increased in obesity. Moreover, leucocytes from obese patients produce higher amounts of visfatin compared with lean subjects, and specifically, granulocytes and monocytes are the major producing cells [137, 138]. Macrophages have been described as a source for visfatin production too [139].

It is supposed that visfatin has insulin mimetic properties; however, the role of this adipokine in glucose metabolism is still unclear [136, 140]. Visfatin is upregulated in models of acute injury and sepsis [141], and its synthesis is regulated by other factors such as glucocorticoids, TNF-*α*, IL-6, and growth hormone (GH). Moreover, visfatin has been shown to induce chemotaxis and the production of IL-1*β*, TNF-*α*, and IL-6 in lymphocytes [138].

## 9. Visfatin and Atherosclerosis

The role played by visfatin in atherosclerosis is still confused, but some studies recognize the involvement of this adipokine in atherosclerotic processes (Figure 1). Serum visfatin concentrations were increased in metabolic syndrome patients with atherosclerotic plaques compared with those without carotid atherosclerosis [142]. Moreover, visfatin expression was found to be increased in symptomatic plaques, while asymptomatic plaques presented lower visfatin expression [143]. Recently, it has been described that visfatin pericoronary fat expression was positively correlated with coronary atherosclerosis [144], in addition CRP and the atherogenic small dense low-density lipoprotein subclasses (sdLDL-C) levels were increased in individuals with higher visfatin levels [145]. All of these data suggest that visfatin develops certain actions in the progression of atherosclerosis, probably related to the fact that visfatin acts as an inflammatory mediator.

*In  vitro* experiments support a proinflammatory role of visfatin. This adipokine induces MCP-1 expression in human endothelial cells via NF-*κ*B and PI3Kinase [146]. In line with this, macrophage foam cells from coronary atherosclerotic lesions produce visfatin, and this is able to enhance inflammatory factors synthesis such as IL-8, TNF-*α*, or MMP-9 in the monocytic cell line THP-1 and in PBMCs [143]. These results indicate strong proinflammatory effects of visfatin, which could be related with atherogenesis and plaque destabilization.

Another study reveals that visfatin could improve endothelial function by increasing eNOS expression [147].

## 10. Visfatin and Rheumatic Diseases

Serum visfatin levels were also increased in RA patients compared with healthy controls [39, 112, 148]. This adipokine has important proinflammatory and catabolic roles in RA pathogenesis, and it is now being intensively studied as a potential target in this illness. In fact, serum and synovial visfatin concentrations were associated with the degree of inflammation, clinical disease activity, and with increased radiographic joint damage [112, 149, 150]. Although in a study that included RA patients with severe disease undergoing anti-TNF-*α* infliximab therapy, visfatin levels were not associated with inflammation or metabolic syndrome and infliximab infusion did not show significant changes in visfatin levels [151], another study showed that prolonged anti- TNF-*α* treatment may reduce visfatin levels [151, 152]. Brentano et al. reported an interesting study, in which high levels of visfatin were observed in the synovial lining layer and at sites of cartilage loss [149]. In addition, the authors demonstrate that visfatin induced IL-6, IL-8, MMP-1, and MMP-3 production in RA synovial fibroblasts as well as IL-6 and TNF-*α* in monocytes. Notably, visfatin knockdown in RA synovial fibroblasts significantly reduced the synthesis of IL-6, IL-8, MMP-1, and MMP-3 [149].

Other authors identified visfatin as a key component of the inflammatory processes leading to arthritis, because visfatin inhibition significantly reduced inflammation, cartilage damage, and the severity of arthritis in a collagen-induced arthritis animal model [153]. Moreover, the inhibition of this adipokine reduced the circulating levels of TNF-*α* [153]. Anyway, the mechanisms by which visfatin exerts its proinflammatory and catabolic functions in the arthritic joint are not well understood, therefore, the use of visfatin as a therapeutic target needs to be studied more in deep.

Visfatin is encoded by the NAMPT gene. Studies on the potential influence of functional NAMPT gene polymorphisms in the risk of cardiovascular disease of RA were conducted. However, no significant association of NAMPT rs9770242 and rs59744560 polymorphisms with disease susceptibility and cardiovasculary risk in patients with RA was observed [154].

At cartilage level, human OA chondrocytes produce visfatin, and similar to IL-1*β*, visfatin is able to enhance the synthesis of prostaglandin E_2_ [155]. This adipokine also increases the expression of ADAMTS 4, ADAMTS5, MMP-3, and MMP-13, which are very relevant cartilage degradative enzymes [155]. To note, OA patients had higher synovial fluid visfatin concentrations, which are correlated with degradation biomarkers such as collagen type II and aggrecan [156]. Taken together, these data indicate that visfatin develops catabolic functions at cartilage level, and it could play an important role in the pathophysiology of OA.

Studies performed in SLE and AS patients present conflicting results. Some authors determined higher visfatin levels in SLE patients than in healthy controls [73], but others did not found any variation between patients and controls [157]. Similarly, there was no association between visfatin levels and disease activity in both SLE and AS [77, 157].

## 11. Resistin

Resistin, known as adipocyte-secreted factor (ADSF) or found in inflammatory zone 3 (FIZZ3), was discovered in 2001 and was proposed as potential link between obesity and diabetes [158]. It was secreted by adipose tissue but has been found also in macrophages, neutrophils, and other cell types. Serum resistin levels increase with obesity in mice, rats, and humans [159, 160]. Resistin belongs to a family of resistin-like molecules (RLM) with distinct expression patterns and biological effects [161].

In animal models, resistin promotes insulin resistance, while the evidence for this effect in human is less clear [158, 162]. Also, it was observed that resistin production is restricted to adipocytes in mice, while in humans it is mainly derived from circulating monocytes and macrophages [163].

## 12. Resistin and Atherosclerosis

Increasing evidence indicates that resistin might play important regulatory roles apart from its role in insulin resistance and diabetes, in a variety of biological processes such as atherosclerosis and cardiovascular diseases (Figure 1). Several studies suggested that resistin was involved in pathological processes, leading to CVD including inflammation, endothelial dysfunction, thrombosis, angiogenesis, and smooth muscle cell dysfunction [164, 165]. Several studies have showed that CVD is accompanied by changes in serum resistin levels, including [166]. Moreover, a similar study demonstrated a significant increase in plasma resistin levels in patients with unstable angina when compared with patients with stable angina or control patients [167]. Resistin levels were elevated in ACS, which has been hypothesized to be due to release of resistin from atherosclerotic plaque during plaque rupture [168]. In addition, the group of Jung has showed that macrophages infiltrating atherosclerotic aneurysms were able to secrete resistin, which in turn, affects endothelial function and vascular smooth muscle cell migration, thus, contributing to atherogenesis [169]. Resistin also might be involved in the maintenance of epithelial cell barrier function, a physical barrier between blood and the arterial wall. In fact, it has been reported that high concentrations of resistin generated in conditional media from epicardial adipose tissue of patients with ACS, increase endothelial cell permeability [170]. Very recently, a novel role of resitin has been described in modulating serum low-density lipoproteins and, thereby, atherosclerotic CVDs in obese humans [171].

## 13. Resistin and Rheumatic Diseases

There are several demonstrations that resistin may be involved in the pathogenesis of RA. Increased levels of this adipokine it have previously been observed in synovial fluid from patients of rheumatoid arthritis (RA) compared to patients with noninflammatory rheumatic disorders [110]. Resistin may be a significant mediator in the inflammatory process of RA. In fact, serum resistin levels are associated with disease activity and acute phase reactants, including C-reactive protein and IL-1Ra antagonizing IL-1*β* [172, 173]. However, resistin-RTN rs1862513 polymorphism was not found to be a genetic risk factor for both clinically evident cardiovascular disease and subclinical atherosclerosis in a large series of patients with RA [174].

Resistin has been found in the plasma and synovial fluid of RA patients, and injection of resistin into mice joints induces an arthritis-like condition, with leukocyte infiltration of synovial tissues, hypertrophy of the synovial layer, and pannus formation [173, 175]. Bokarewa et al. have showed that resistin induces and is induced by several proinflammatory cytokines, such as TNF-*α* or IL-6, in peripheral blood mononuclear cells, via NF-*κ*B pathway, indicating that resistin can increase its own activity by a positive feedback mechanism [175]. This group has recently demonstrated that resistin utilizes IGF-1R pathway in RA synovial [176].

Increased serum resistin in patients with rheumatoid arthritis correlated with both C-reactive protein (CRP) and DAS28, suggesting a role of this adipokine in the pathogenesis of rheumatoid arthritis [173]. Gonzalez-Gay et al. have confirmed this association between laboratory markers of inflammation, particularly CRP and resistin levels and have showed that anti-TNF-alpha therapy results in a rapid reduction of serum resistin levels in patients with RA [177].

Recent experimental data suggest that resistin, in the presence of dentritic cells, might induce the expansion of functional regulatory T cells [178].

The proinflammatory profile of resistin, together with its association with obesity, suggests that this adipokine might be another potential mediator that links OA with inflammation and obesity.

In addition, resistin has a role as a marker of inflammation in other rheumatic diseases, such as systemic lupus erythematous (SLE) [179]. In fact, Almehed et al. have demonstrated a positive correlation between serum resistin levels, inflammation, bone mineral density, and renal functions in patients with SLE [180].

In a very recent study, higher serum resistin levels have been reported in patients with AS compared to healthy subjects giving clues that resistin could have also a role in the pathogenesis of AS [181].

## 14. Conclusions

Adipose tissue-derived factors, called adipokines, are now considered to play multiple and relevant roles in the body, including a complex adipokine-mediated interaction among white adipose tissue, cardiovascular disorders, and rheumatic diseases. The chronic increase of the inflammatory tone is generally associated with an increased risk for the development of cardiovascular diseases, and the proinflammatory environment presented in rheumatic diseases contributes to the increase of severe cardiovascular disorders. In addition, the inflammatory functions exerted by adipokines in certain rheumatic diseases could explain some of their associated cardiovascular comorbidities, suggesting a potential therapeutic role for these molecules.

Anyway, the main causes of abnormal fat mass accumulation and adipokine dysfunction are bad nutritional and lifestyle habits, such as overeating and physical inactivity. Therefore, the first therapeutic approach for cardiovascular disorders in rheumatic diseases should be the correction of these bad habits.

 In summary, modification in the lifestyle, as well as other therapeutic interventions leading to reduce fat mass, and its associated dysfunction might improve cardiovascular mortality in patients with rheumatic diseases.
